# Investigating COVID-19 Pandemic Effects on Acute Pancreatitis Development—From the Perspective of Alcohol Sales (Consumption) in a Japanese Regional Hospital

**DOI:** 10.3390/healthcare11202769

**Published:** 2023-10-19

**Authors:** Fumi Sakuma, Akira Yamamiya, Yoko Abe, Kazunori Nagashima, Takahito Minaguchi, Ken Kashima, Yasuhito Kunogi, Koh Fukushi, Yasunori Inaba, Takeshi Sugaya, Keiichi Tominaga, Kenichi Goda, Atsushi Irisawa

**Affiliations:** Department of Gastroenterology, School of Medicine, Dokkyo Medical University, 880 Kitakobayashi Mibu, Tochigi 321-0293, Japan; sakuma-f@dokkyomed.ac.jp (F.S.); y-abe808@dokkyomed.ac.jp (Y.A.); n-kazu@dokkyomed.ac.jp (K.N.); takahito@dokkyomed.ac.jp (T.M.); ken-k@dokkyomed.ac.jp (K.K.); ykunogi@dokkyomed.ac.jp (Y.K.); d-fuku-k@dokkyomed.ac.jp (K.F.); inaba911@dokkyomed.ac.jp (Y.I.); t-sugaya@dokkyomed.ac.jp (T.S.); tominaga@dokkyomed.ac.jp (K.T.); goda@dokkyomed.ac.jp (K.G.); irisawa@dokkyomed.ac.jp (A.I.)

**Keywords:** acute pancreatitis, alcohol consumption, COVID-19, state of emergency declaration

## Abstract

[Aim and Background] People’s lifestyles changed considerably due to the coronavirus disease 2019 (COVID-19) pandemic. The number of patients with acute pancreatitis (AP) can be expected to decrease as alcohol consumption decreases. This study was conducted to assess COVID-19 pandemic effects on AP patients in a Japanese regional hospital. [Methods] Based on the first and second states of emergency declarations in Tochigi Prefecture, the survey periods were set as follows: period A, 16 April–14 May; period B, 15 May–13 January; period C, 14 January–7 February; and period D, 8 February–15 April. Using data acquired in 2017, 2018, 2019, and 2020, we retrospectively reviewed the number of patients admitted to our hospital with a diagnosis of AP, and their clinical characteristics. [Results] According to a National Tax Agency survey, the average alcohol sales per adult in Tochigi Prefecture were 71.3 L in 2017 before the pandemic, and 64.0 L in 2021 under the pandemic. The number of AP patients in 2020 was 38% lower than in 2017. Comparing 2017 with 2020, the number of alcoholic AP patients was lower in 2020 (*p* = 0.007). [Conclusions] The findings suggest that COVID-19-pandemic-related lifestyle changes contributed to the decrease in AP patients.

## 1. Introduction

Coronavirus disease 2019 (COVID-19), which began in Wuhan, China in 2019, has spread around the world. On 16 January 2020, the first infected person was confirmed in Japan. After that, the infection spread, and the first state of emergency was declared on 7 April 2020. It has been confirmed that the infection will have spread eight times by the winter of 2023. As of summer 2023, the spread of infection is converging. When the infection first began to spread, the first of four emergency declarations was issued in Japan. Thereafter, people’s lifestyles changed considerably [1]. Many schools closed and classes for students changed from face-to-face to online. Companies took special and unprecedented measures such as encouraging employees to work from home, staggered working hours, and ensuring social distancing. Conversation without a mask increases the risk of infection, so restrictions on activities at night, especially when alcohol is involved (restraint and restrictions on drinking parties, etc.), were observed. Restaurants were closed, and people were less likely to dine in restaurants. In Japan, the consumption of alcoholic beverages reportedly decreased because of the state of emergency declaration [2]. Therefore, the number of patients with diseases caused by alcohol was presumably affected. The number of patients with acute pancreatitis, which is well known to be caused mostly by alcohol consumption, can be expected to decrease as alcohol consumption decreases. Moreover, medical institutions were compelled to deal with COVID-19 patients during the spread of infection, consequently affecting systems providing ordinary medical care [3,4,5,6,7,8,9,10]. Some hospitals restricted elective surgeries, routine outpatient visits, and emergency department visits. On the patient side, even if they had symptoms, some avoided visiting the hospital for fear of being infected with COVID-19. Given this background, we have investigated the COVID-19 pandemic effects on the number of cases of AP onset at our regional university hospital in Japan, with the aim of clarifying the actual situation.

## 2. Materials and Methods

### 2.1. Study Design

This observational study was conducted at a university hospital (1195 beds) in Tochigi Prefecture, Japan. The Medical Ethics Committee of our institution (Dokkyo Medical University Hospital, R-48-3J) approved this study, which has been registered with the University Hospital Medical Information Network (UMIN) Clinical Trials Registry (000059228). A means to opt out was provided to patients instead of informed consent: research subjects were notified and were granted the opportunity via our website to refuse publication of their research information.

The primary endpoint was examination of changes in the number of AP patients under the COVID-19 pandemic at a regional hospital. The secondary endpoint was the relation between alcohol consumption and alcoholic AP, changes in AP severity, and sex differences of AP patients under the COVID-19 pandemic.

### 2.2. Japan’s State of Emergency Declarations and Infection Countermeasures

In April 2020, the Japanese government issued a state of emergency declaration in response to the spread of the new coronavirus infection [1]. (1) The frequency of pneumonia was recognized as considerably higher than that of seasonal influenza virus infection (2) In many cases, the infection route was not identifiable (3) The rapid increase in the number of infected people was confirmed. The government requested the public’s cooperation in necessary measures to prevent infection (wearing masks, practicing cough etiquette, washing hands, gargling, etc.), refraining from going out, and restricting the use of facilities. The medical care system became more tightly controlled. Therefore, a risk of severe damage was posed to the lives and health of people. The measures strongly affected people’s lives and affected the national economy because of the rapid spread nationwide. A nationwide state of emergency was issued during the following periods: first state of emergency, 7 April 2020–25 May 2020; second state of emergency, 8 January 2021–21 March 2021; third state of emergency, 25 April 2021–20 June 2021; and fourth state of emergency, 12 July 2021–30 September 2021. Based on these periods, the timing of the emergency declarations differed among prefectures. States of emergency for Tochigi Prefecture were issued: first state of emergency, 16 April 2020–14 May 2020; second state of emergency, 14 January 2021–7 February 2021; and third state of emergency, 20 August 2021–30 September 2021. During the first and second emergency periods, restaurants were restricted to shortened business hours until 8:00 p.m., with liquor served until 7:00 p.m. For the third emergency declaration, the government asked restaurants providing alcohol and karaoke facilities to close. If the restaurants provided no alcohol, then the government requested a closing time of 8:00 p.m. as a priority measure to prevent the virus’s spread. A system was established in which municipalities would pay cooperation funds to restaurants that cooperated with the government’s requests. These restrictions were eased after the emergency declaration.

### 2.3. Patients

Based on the first and second state of emergency declarations in Tochigi Prefecture, the survey periods were set as follows: period A, 16 April–14 May; period B, 15 May–13 January; period C, 14 January–7 February; and period D, 8 February–15 April day. Using data from 2017, 2018, 2019, and 2020, we retrospectively examined the number of patients and the clinical characteristics of those admitted to our university hospital for treatment with a diagnosis of AP.

### 2.4. Japanese Criteria for Assessing AP Severity

To evaluate the prognostic factors and CE-CT grading for AP, the Japanese criteria for assessing the severity of AP developed by the Research Committee of Intractable Diseases of the Pancreas (Ministry of Health, Labour and Welfare) were used (Supplementary Table S1) [11,12]. Prognostic factors comprised the following nine items: (1) base excess (BE) ≤ 2–3 mEq/L or shock (systolic blood pressure < 80 mmHg), (2) PaO_2_ ≤ 60 mmHg, (3) blood urea nitrogen ≥ 40 mg/dL (or creatinine ≥ 2.0 mg/dL) or oliguria after fluid replacement, (4) lactic dehydrogenase (LDH) 2 times the upper limit of normal, (5) platelet count ≤ 100,000/mm^3^, (6) serum calcium ≤ 7.5 mg/dL, (7) C-reactive protein ≥ 15 mg/dL, (8) number of positive measures in the systematic inflammatory response syndrome (SIRS) criteria ≥ 3, and (9) age ≥ 70 years. Patients who satisfied more than three of those nine items above were inferred as having severe AP. The CE-CT grade is a classification for severity assessment made by the combination of two factors: the degree of extrapancreatic progression of inflammation and the extent of low enhanced pancreatic parenchyma (LEPP). Extrapancreatic progression of inflammation up to the anterior pararenal space was 0 point, up to the root of the mesocolon was 1 point, and beyond the lower pole of the kidney was 2 points. LEPP, which localized in each segment or only surrounding the pancreas, was 0 point, extended to two segments was 1 point, and occupied two entire segments or more was 2 points. These points were totaled, and a score of 2 or more was considered severe (CT grade 2, 3) [11,13].

### 2.5. Statistical Analysis

Statistical analyses were performed using statistical analysis software (SPSS ver. 27.0; SPSS Inc., Chicago, IL, USA). Data were analyzed using the Mann–Whitney U test and χ^2^ test. Differences for which *p* < 0.05 were inferred as significant.

## 3. Results

### 3.1. Changes in Alcohol Consumption under the COVID-19 Pandemic

Changes in alcohol consumption before and under the COVID-19 pandemic are presented in Table 1. According to a National Tax Agency survey [2], the nationwide alcohol sales (consumption) per adult in Japan were 80.5 L in 2017 before the pandemic and 74.3 L in 2021 during the pandemic. These data represent a reduction of about 7%. The alcohol sales (consumption) per adult in Tochigi Prefecture was 71.3 L in 2017 before the pandemic, and 64.0 L in 2021 during the pandemic. These data represent a reduction of about 10%.

### 3.2. Number of AP Patients and Etiology

**Figure 1:**
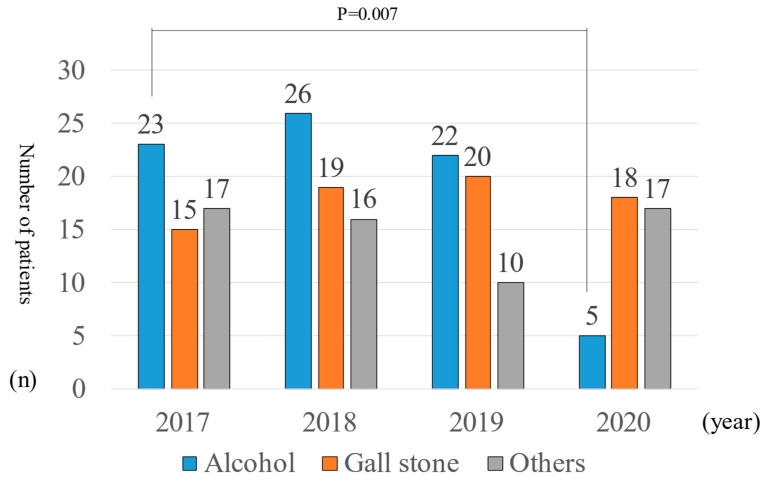
Number of AP patients and etiology.

The total numbers of AP patients and the associated etiologies are presented, respectively, in Figure 1. The total number of AP patients (alcoholic AP, gallstone AP, others) were the following. The number of AP patients in 2017 was 55 (23:15:17) (period A, 6 (3:2:1); B, 35 (14:9:12); C, 5 (2:2:1); D, 9 (4:2:3)). The number of AP patients in 2018 was 61 (26:19:16) (period A, 9 (4:3:2); B, 41 (17:12:12); C, 1 (0:1: 0); D: 10 (5:3:2)). The number of AP patients in 2019 was 52 (22:20:10) (period A, 5 (3:2:0); B, 36 (16:14:6); C, 3 (1:1:1); D: 8 (2:3:3)). The number of AP patients in 2020 was 34 (5:18:11) (period A, 4 (2:1:1); B, 24 (3:13:8); C, 3 (0:3: 0); D: 3 (0:1:2)). The number of AP patients in 2020 was therefore 38% lower than in 2017. A comparison of 2017 and 2020 shows that the total number of alcoholic AP patients was significantly lower in 2020 (*p* = 0.007). Particularly, the alcoholic AP patients were significantly fewer from January 2020 onwards.

### 3.3. Number of Patients with Severe AP 

**Figure 2:**
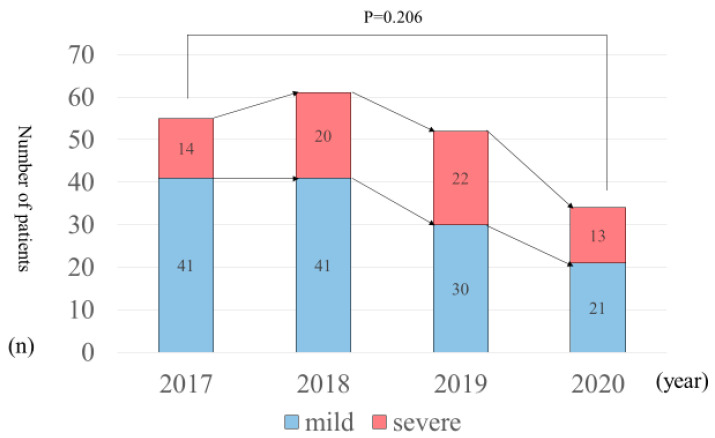
Number of patients with severe AP.

The total numbers of patients with severe AP are presented in Figure 2. The numbers of severe AP (severe: mild) patients were the following. The numbers of severe AP cases in 2017 were 14:41 (period A, (3:3); B, (7:28); C, (2:3); D, (2:7)). The numbers of severe AP cases in 2018 were 20:41 (period A, (4:5); B, (11:30); C, (1:0); D, (4:6)). The numbers of severe AP cases in 2019 were 22:30 (period A, (5:0); B, (8:28); C, (3:0); D, (6:2)). The numbers of severe AP cases in 2020 were 13:21 (period A, (2:2); B, (10:14); C, (0:3); D, (1:2)). A comparison of 2017 and 2020 reveals no significant differences between the two groups in terms of severity of AP (*p* = 0.206).

### 3.4. Sex Differences of AP Patients

**Figure 3:**
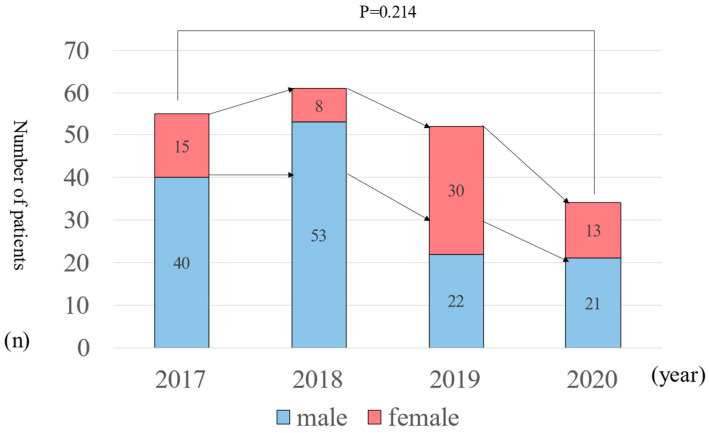
Sex differences of AP patients.

Sex differences related to alcohol metabolism are known. Based on this knowledge, we also investigated sex differences in the onset of acute pancreatitis. The sex differences of AP patients are presented in Figure 3. Sex differences of AP patients (male: female) were found to be the following. In 2017, findings were 40:15 (period A, (5:1); B, (24:11); C, (5:0); D, (6:3)). In 2018, findings were 53:8 (period A, (8:1); B, (36:5); C, (1:0); D, (8:2)). In 2019, findings were 36:16 (period A, (5:0); B, (22:14); C, (3:0); D, (6:2)). In 2020, findings were 36:13 (period A, (3:1); B, (13:11); C, (2:1); D, (3:0)). A comparison of 2017 and 2020 shows no significant differences between the two groups in terms of sex differences (*p* = 0.214).

## 4. Discussion

Acute pancreatitis is caused by activation of pancreatic enzymes in the pancreatic acinar cells, attributable to some cause [11,14]. Consequently, pancreatic autolysis occurs. Alcohol and gallstones are the two most common causes of acute pancreatitis in Japan. Alcoholic acute pancreatitis is generally more common in men. According to data from the Ministry of Health, Labour and Welfare of Japan, the total number of AP patients is decreasing year by year [15]. According to a report by the National Tax Agency, the consumption of alcoholic beverages in Japan peaked in 1992, and has been decreasing year by year ever since [2]. Today, COVID-19, which has been spreading since 2019, is raging worldwide. The eighth wave of the disease arrived in Japan in January 2023. The government of Japan declared states of emergency four times to prevent the spread of COVID-19 infection [1]. The declaration of the state of emergency drastically changed the way of life of the people. Particularly, the government requested restaurants to close for extended periods of time, shorten their hours of operation, and prohibit the serving of alcoholic beverages. People refrained from eating out to prevent infection, and opportunities to eat with large groups disappeared. As a result, many restaurants went out of business. Our facility is located in Tochigi Prefecture, which is more rural than Japan’s capital city of Tokyo. Compared with Tokyo, the infected people were few. Therefore, only three emergencies were declared because of the spread of COVID-19. We hypothesized that changes in people’s drinking habits attributable to the emergency declaration would lead to changes in the number of AP cases.

Drinking habits are recognized worldwide. Alcohol is not merely a drink; it plays a variety of roles, giving people a feeling of release, eliminating fatigue, instilling a cheerful mood, and lubricating human relationships. The harmful effects of excessive drinking are also well known. According to the World Health Organization’s “Global Status Report on Alcohol and Health 2018” [16], alcohol is involved in more than 200 health difficulties. Alcohol causes more deaths than tuberculosis. Diabetes, HIV/AIDS, and other health problems related to alcohol are responsible for three million deaths worldwide. In recent years, because of the COVID-19 pandemic, attention has come to be focused on drinking alcohol and COVID-19 infection. Alcoholic beverages are often used to cope with stress and anxiety, and it has been reported internationally that increased alcohol consumption and exacerbation of drinking problems because of the novel coronavirus epidemic have become an important public health issue [17,18,19].

During the SARS pandemic in 2003, among more than 800 Hong Kong residents, 4.7% of men and 14.8% of women who had been drinking habitually reported increased drinking one year after the SARS pandemic ended [20]. Furthermore, health workers such as those in Beijing who worked in isolation wards or high-risk wards had an approximately 1.5 times higher risk of alcohol dependence three years after the end of the SARS pandemic than health workers not working in those wards [21]. In Canada, hospital admissions for alcohol-related mental and behavioral disorders and alcoholic AP increased significantly during the COVID-19 pandemic [6]. In the United States, reportedly when the COVID-19 pandemic began in 2020, alcohol-related deaths increased by 26% compared with those in 2019, to 99,017. In addition, during the isolation period, binge drinking increased among male healthcare workers, and habitual drinking increased among females [22]. For data limited to New York, during the first peak of COVID-19 (1 March 2020–31 May 2020), the number of patients with alcohol withdrawal increased, but the number of alcohol-related illnesses decreased [23]. Support for sobriety has decreased, revealing a worsening of alcohol consumption and drinking-related difficulties [24]. According to a review of alcohol drinking and the COVID-19 pandemic, factors that have been shown from earlier studies to increase the risk of drinking include heavy drinking before the pandemic, high levels of anxiety and depression, unemployment, and reduced social ties [25]. In response to concerns about worsening global drinking problems due to the COVID-19 pandemic, the WHO has also issued recommendations related to alcohol consumption [15]. By contrast, Japan is changing differently compared with other countries. According to a National Tax Agency survey [2], nationwide average alcohol sales (consumption) per adult in Japan were 80.5 L in 2017 before the pandemic and 74.3 L in 2021 during the pandemic. Regarding alcohol consumption at restaurants, according to the Ministry of Internal Affairs and Communications household budget survey, the average consumption expenditure per household in December 2021 was 55.9% lower than in 2019. This decreased alcohol consumption is thought to be caused by a decrease in “drinking out”, attributable to fewer opportunities to dine out and to the effects of retail store closures during the pandemic. Itoshima et al. reported a 12-fold increase in the number of patients hospitalized for alcoholic liver injury and pancreatitis [26]. In addition, an online survey conducted in 2021 with screening tests revealed that 10.4% of men and 40% of women were suspected to have alcohol dependence (AUDIT score of 15 or higher) [27]. Even with this reduction in overall drinking, it is possible that some specific populations, such as persons with alcoholism, were exacerbating their drinking-related difficulties. The aforementioned Ministry of Internal Affairs and Communications Household Budget Survey showed that the amount spent on “Chu-hi cocktails” increased by 32%, indicating an increase in the amount of alcohol consumed at home, partly because of the spread of online drinking parties [2].

For this study, the number of AP patients in 2020 was 31% lower than in 2017. In Japan, people refrained from visiting hospitals to avoid infection during the COVID-19 pandemic. According to an announcement by the Ministry of Health, Labour and Welfare of Japan, the number of patients decreased by 8.5% in 2020 compared with the previous year [28]. The same situation has been reported in other countries. To prevent the spread of COVID-19, health care providers delayed appointments or moved to telemedicine. In Thailand, the number of daily emergency department visits decreased significantly during the lockdown due to the COVID-19 pandemic [4]. Similarly, another study conducted in Melbourne, Australia, found that the number of emergency department visits decreased significantly during the COVID-19 pandemic [5]. In Canadian hospitals with emergency departments, the number of inpatients in both internal medicine and surgery departments decreased significantly during the COVID-19 pandemic [6]. Thus, we think that the decrease in the number of AP patients during the COVID-19 pandemic was partly due to the decrease in the number of outpatient visits. However, this decreased number of AP patients may not represent the true number of AP patients during the COVID-19 pandemic. Although there are individual differences, the symptoms of AP often depend on the severity. In cases of mild AP, the abdominal pain is often mild. Some of these patients may have been followed up at home without visiting the hospital. The patients with mild symptoms refrained from visiting the hospital not only for AP but also for stroke and acute coronary syndrome [10,29]. Fortunately, the fatality rate for mild cases of AP in Japan is 0.5% [30], and there are many cases in which the condition recovers even without hospital treatment. However, this study reported that the number of alcoholic AP patients has decreased considerably since January 2020, particularly. Reportedly, alcohol sales (consumption) per adult nationwide have decreased by 10% in Japan and by 7% in Tochigi Prefecture [2]. The findings suggest that lifestyle changes and decreasing alcohol consumption caused by the COVID-19 pandemic might have contributed directly to the decrease in AP patients. From these results, among people at risk for alcoholic AP, a 10% reduction in alcohol consumption might reduce the risk of developing AP. In Australia, AP admissions in 2020 decreased by nearly 20% compared with the same four months in 2019 [7]. Even after the end of the COVID-19 pandemic, if lifestyle guidance can be provided to reduce the amount of alcohol consumed by each person, then it might lead to a further decrease in the number of AP patients. 

In addition, we considered regional disparities in alcohol consumption. Alcohol sales (consumption) per adult in Tochigi Prefecture, where our facility is located, decreased by 10% before and after the COVID-19 pandemic [2]. This result shows a larger decrease than the national average. Because the main transportation in Tokyo is by rail, it is easy to leave one’s home and drink at a restaurant. By contrast, in Tochigi Prefecture, which is more rural than Tokyo, the main transportation is by private passenger car. Drinking opportunities at restaurants and other venues are therefore fewer than in Tokyo. We regard this reason as leading to a decrease in alcohol consumption. In addition, the population of Tochigi Prefecture is approximately 1.9 million people, and the proportion of the population between the ages of 15 and 64 is approximately 58%, and 65 years of age or older is approximately 30% [31]. On the other hand, the population of Tokyo is approximately 9.77 million people, and the proportion of the population between the ages of 15 and 64 is approximately 66%, and 65 years of age or older is approximately 23% [32]. Compared with Tokyo, the population ratio of Tochigi Prefecture is that there are fewer university students and working aged people who often drink alcohol, and there are many elderly people who have fewer opportunities to drink alcohol. This fact also leads to the result that alcohol sales (consumption) per adult in Tochigi Prefecture is lower than the national average. When drinking at restaurants, large amounts of alcohol are expected to be consumed by the large number of people attending, due to the short amounts of time and the unlimited amount of alcohol available with so-called Nomihodai (all-you-can-drink) menu options. However, it is expected that people who drink at home consume alcohol alone or with a small group of family members over a long time. Changes in drinking habits might contribute to the number of cases of AP too. In 2018, Kawaida et al. reported on the influence of the use of such Nomihodai systems on alcohol consumption [33]. The amount of drinking was increased during Nomihodai sessions, compared with non-use states: 1.8-fold among men, and 1.7-fold among women. Heavy episodic drinking occurred only in Nomihodai situations. Lifestyle changes associated with the spread of coronavirus infection have also probably affected drinking styles. Although the household survey examines the amount spent on alcoholic beverages, it remains unclear how much of the purchased alcoholic beverages are actually consumed at home. However, it can be expected that few people drinking alcohol alone or with family members at home consume as much as they drink during Nomihodai situations. According to a report by the National Tax Agency survey in 2020 [2], alcohol sales (consumption) by category decreased significantly for beer (77%) and whiskey (87%). On the other hand, alcohol sales (consumption) of sweet fruit drinks (110%), spirits (109%), and liqueurs (107%) increased. In other words, these results demonstrate that opportunities to drink at restaurants have decreased and opportunities to drink at home have increased. Although there are regional differences, given this background for this study, it can be expected that the number of alcoholic AP patients decreased.

In this study, a comparison of 2017 and 2020 showed no significant differences between AP patients in terms of sex differences. Females are more susceptible to the effects of alcohol than male due to differences in their biological characteristics [34]. Alcohol consumption is said to be influenced by stress such as unemployment and social isolation during lockdown [18]. In fact, during the COVID-19 pandemic, the unemployment rate in Japan increased, especially among women [35]. Now that the effects of COVID-19 are fading, the unemployment rate is decreasing. However, the influence of such social backgrounds on the number of alcoholic AP patients from the perspective of sex differences cannot be denied, and this should be considered as a topic for future investigation.

The limitations of this study are its single-center focus, its retrospective design, and its small number of patients. In addition, under the COVID-19 pandemic, hospitalization restrictions were also implemented at our hospital to prevent the spread of infection. Our hospital, although a nationally certified special treatment hospital, is in Tochigi Prefecture, which has fewer hospitals than large cities. Therefore, during the COVID-19 pandemic, we treated many patients, not only within the prefecture but also outside the prefecture. All cases that requested admission to our hospital with a diagnosis of AP were able to be admitted. In fact, the numbers of gallstone AP patients and severe AP patients assessed for this study remained unchanged before and after the COVID-19 pandemic. Therefore, this limitation does not affect the idea that the decrease in the number of patients with acute pancreatitis is attributable to decreased alcoholic AP associated with drinking habits. During the summer of 2023, due to changes in the classification of the Infectious Diseases Act, regulations were significantly relaxed. Economic activity regained vigor, and people are reverting to their lifestyles they enjoyed before the COVID-19 pandemic. The number of patients including AP at our hospital is also increasing. Based on the results obtained from this study, we believe that it is necessary to conduct a prospective study on the relationship between alcohol consumption and the number of cases of acute pancreatitis, and to disseminate this information to the world. Even with this much progress in medical care, medical professionals believe that it is an important duty to control acute pancreatitis firmly from a lifestyle perspective, where many patients lose their lives or suffer from late complications such as walled-off necrosis. This will also lead to reduced medical costs. 

## 5. Conclusions

Although drinking alcohol is not the only cause of acute pancreatitis, from our study, the COVID-19 pandemic caused lifestyle changes associated with decreased alcohol consumption, which might have influenced the development of AP. Future multicenter studies must be conducted to examine the effects of regional and sex differences. Based on this, we think it would be good if guidelines for appropriate lifestyle guidance could be established.

## Figures and Tables

**Table 1:** Changes in alcohol consumption per adult in Japan.

	In 2017	In 2018	In 2019	In 2020	In 2021
Nationwide	80.5 L	79.3 L	78.1 L	75.0 L	74.3 L
Tokyo	111.6 L	107.8 L	105.0 L	95.5 L	96.6 L
Tochigi	71.3 L	69.2 L	67.1 L	65.3 L	64.0 L

## Data Availability

No new data were created or analyzed in this study. Data sharing is not applicable to this article.

## Supplementary Materials

The following supporting information can be downloaded at: https://www.mdpi.com/article/10.3390/healthcare11202769/s1, Table S1. Japanese severity scoring system for acute pancreatitis (Ministry of Health, Labor and Welfare of Japan, 2008 revision).
