# Supplementary material for: Investigating COVID-19 Pandemic Effects on Acute Pancreatitis Development—From the Perspective of Alcohol Sales (Consumption) in a Japanese Regional Hospital
Source: Healthcare (Basel). 2023 Oct 19;11(20):2769. doi: 10.3390/healthcare11202769 (PMC10606278; doi:10.3390/healthcare11202769)
Supplement: Supplementary file 1 [file healthcare-11-02769-s001.zip › healthcare-2592909-supplementary.pdf]

Supplementary Table S1. Japanese severity scoring system for acute pancreatitis (Ministry of Health, Labor and Welfare of Japan, 2008 revision)

Prognostic factors (1 point for each factor)

1. Base excess ≤ –3 mEq /L or shock (systolic blood pressure <80 mmHg)
2. P_aO_2 ≤60 mmHg (room air) or respiratory failure (requiring ventilatory management)
3. BUN ≥ 40 mg/dl or creatinine ≥ 2.0 mg/dl or oliguria (daily urine output <400 ml even after intravenous fluid resuscitation)
4. Lactate dehydrogenase ≥2 ULN
5. Platelet count ≤100 000/ mm^3
6. Serum Ca ≤7.5 mg/dl
7. C-reactive protein ≥ 15 mg/dl
8. No. positive measures in SIRS criteria ≥ 3
9. Age ≥ 70 y

CT grade based on contrast-enhanced CT

1 Extrapancreatic progression of inflammation

Anterior pararenal space	0 point
Root of mesocolon	1 point
Beyond lower pole of kidney	2 points

2. Low enhanced lesion of the pancreas

The pancreas is conveniently divided into three segments (head, body, and tail)

Localized in each segment or only surrounding the pancreas	0 point
Extends to two segments	1 point
Occupies two entire segments or more	2 points

1 + 2 = total score

Total score = 0 or 1	Grade 1
Total score = 2	Grade 2
Total score ≥3	Grade 3

Assessment of severity

If the prognostic factors score is ≥ 3 or CT grade is ≥ 2, the disease is graded as 'severe'.

The systemic inflammatory response syndrome (SIRS) criteria include body temperature >38° C or <36° C , heart rate >90 bpm, respiratory rate >20 breaths/min or P_aCO_2 ≤32 torr, and white blood cell counts > 12 000 cells/mm^3, <4000 cells/mm^3, or >10% immature (band) forms.

BUN, blood urea nitrogen; ULN, upper limit of normal; CT, computed tomography.
